# High-Sensitivity C-Reactive Protein Modifies P-Wave Terminal Force in Lead V1-Associated Prognosis in Acute Ischemic Stroke or TIA Patients

**DOI:** 10.3390/jcm12052031

**Published:** 2023-03-03

**Authors:** Yueyang Wu, Wei Lv, Jiejie Li, Xiaomeng Yang, Xia Meng, Zixiao Li, Yuesong Pan, Yong Jiang, Hongyi Yan, Xinying Huang, Liping Liu, Xingquan Zhao, Yilong Wang, Hao Li, Yongjun Wang

**Affiliations:** 1 Department of Neurology, Beijing Tiantan Hospital, Capital Medical University, Beijing 100070, China; 2 China National Clinical Research Center for Neurological Diseases, Beijing 100070, China; 3 Advanced Innovation Center for Human Brain Protection, Capital Medical University, Beijing 100070, China; 4 Research Unit of Artificial Intelligence in Cerebrovascular Disease, Chinese Academy of Medical Sciences, Beijing 100070, China; 5 Center for Excellence in Brain Science and Intelligence Technology, Chinese Academy of Sciences, Shanghai 200031, China

**Keywords:** hsCRP, prognosis, PTFV1, stroke

## Abstract

Little is known about the role of high-sensitivity C-reactive protein (hsCRP) in the relationship between P-wave terminal force in lead V1 (PTFV1) and stroke prognosis. We aimed to investigate how hsCRP influences the effect of PTFV1 on ischemic stroke recurrence and mortality. In this study, patients enrolled in the Third China National Stroke Registry, which enrolled consecutive patients who had suffered an ischemic stroke or transient ischemic attack in China, were analyzed. After excluding patients with atrial fibrillation, 8271 patients with PTFV1 and hsCRP measurements were included in this analysis. Cox regression analyses were used to assess the association between PTFV1 and stroke prognosis according to different inflammation statuses stratified by an hsCRP level of 3 mg/L. A total of 216 (2.6%) patients died, and 715 (8.6%) patients experienced ischemic stroke recurrence within 1 year. In patients with hsCRP levels ≥ 3 mg/L, elevated PTFV1 was significantly associated with mortality (HR, 1.75; 95% CI, 1.05–2.92; *p* = 0.03), while in those with hsCRP levels < 3 mg/L, such an association did not exist. In contrast, in patients with hsCRP levels < 3 mg/L and those with hsCRP levels ≥ 3 mg/L, elevated PTFV1 remained significantly associated with ischemic stroke recurrence. The predictive role of PTFV1 towards mortality but not ischemic stroke recurrence differed in terms of hsCRP levels.

## 1. Introduction

Atrial cardiopathy is a term used to describe atrial structural and pathophysiologic changes that can precede atrial fibrillation (AF), which is known to be associated with an increased risk of ischemic stroke and mortality after stroke [1,2]. P-wave terminal force in lead V1 (PTFV1) is a well-investigated atrial cardiopathy marker applied to electrocardiography (ECG). It has been shown to be associated with the risk of incident ischemic stroke and mortality [3,4]. The production of a substrate for thrombus formation and subsequent embolization to the brain has been suggested to account for the risk of stroke correlated with PTFV1 [5]. More importantly, such an association persists even in the absence of AF, and a stronger association of PTFV1 with incident stroke than with incident AF has been revealed [5,6,7,8]. These findings suggest that PTFV1 plays a crucial role in the early screening for the risk of atrial-cardiopathy-related stroke. In contrast, limited data are available regarding the effect of PTFV1 on recurrent stroke in patients without AF.

On the other hand, patients with a high inflammation burden have an increased risk of recurrent stroke and mortality after stroke [9,10,11]. Furthermore, inflammation has also been shown to be involved in a series of pathophysiological abnormalities that cause atrial cardiopathy to progress to stroke [12], such as endothelial dysfunction [13], impaired myocyte function, blood stasis [14], and myocardial remodeling [15]. High-sensitivity C-reactive protein (hsCRP) is a commonly used and accessible inflammatory marker, and whether the role of PTFV1 in recurrence and mortality after stroke varies according to hsCRP level is still unknown.

The Third China National Stroke Registry (CNSR-III) enrolled patients with acute ischemic stroke and transient ischemic attack (TIA) in order to identify prognostic markers and promote the early evaluation and identification of high-risk patients [16]. We used the CNSR-III cohort to determine whether hsCRP levels affect PTFV1-associated ischemic stroke recurrence and mortality in patients with cerebrovascular disease but without AF.

## 2. Materials and Methods

### 2.1. The Study Design and Participants

The CNSR-III was a nationwide, prospective registry developed between August 2015 and March 2018 that enrolled 15166 patients that had suffered ischemic stroke or TIA within seven days after symptom onset. The study included 201 hospitals in 26 provinces and municipalities in China. The prespecified biomarker substudy of the CNSR-III incorporated 171 study sites from which blood samples had been previously collected. Fasting blood samples were obtained within 24 h of admission. The CNSR-III was approved by the Ethics Committee at Beijing Tiantan Hospital and all participating centers. Informed consent was provided by all patients or their legally authorized representatives. The detailed study design and methods of the CNSR-III have been described previously [16].

### 2.2. Baseline Data

The baseline clinical data of patients enrolled in the CNSR-III were collected by trained research coordinators at each institute, and these data included age, sex, body mass index (calculated as weight in kilograms divided by height in meters squared, kg/m^2^), smoking habits, and medical history (stroke, TIA, diabetes, hypertension, dyslipidemia, coronary heart disease, and heart failure). The severity of stroke upon admission was measured using the National Institutes of Health Stroke Scale (NIHSS) score, and baseline leukocyte count as well as medication usage, e.g., antiplatelets, anticoagulants, antihypertensive agents, hypoglycemic agents, and lipid-lowering agents, were also recorded.

### 2.3. Sample Collection and Measurements of hsCRP

The median time of sampling was 55 h (interquartile range, 27–96 h) after the onset of the index event. Serum and plasma specimens were extracted, transported through the cold chain, and subsequently stored at −80 °C in the core laboratory at Beijing Tiantan Hospital. All assays were performed centrally and blindly. The concentrations of plasma hsCRP were measured on Roche Cobas C701 analyzers.

### 2.4. Atrial Cardiopathy Marker

Upon admission, each patient underwent a standard 12-lead ECG with a speed of 25 mm/s and calibration of 10 mm/mV. The ECG was digitalized and amplified to up to 5 times its original size. A well-trained cardiologist blinded to the clinical data of patients measured PTFV1 using digital calipers. The measurements were repeated thrice, and the average values were obtained for analysis. PTFV1 was defined as the duration (ms) times the absolute value of the depth (μV) of downward deflection (terminal portion). Previous studies have demonstrated excellent intra-rater correlations for manual measurements of PTFV1 [7]. To evaluate interrater reliability, a second well-trained cardiologist performed blinded measurements of a random sample of 200 ECGs. The interrater intraclass correlation coefficient was 0.78 (95% CI, 0.67–0.85).

### 2.5. Outcomes and Follow-Up

The evaluated outcomes were all-cause mortality and ischemic stroke recurrence within 1 year. Patients were interviewed face-to-face at 3 months, while trained research coordinators contacted the patients at 6 months and 1 year. Death certificates were obtained either from the relevant hospitals or the local citizen registry. Ischemic stroke recurrence was confirmed through hospital visits. Suspected cases of ischemic stroke recurrence without hospitalization were judged by independent endpoint judgement committee. A detailed description of the follow-up procedure has been published previously [16].

### 2.6. Statistical Analysis

Continuous variables were reported as median with interquartile range and examined using the Mann–Whitney U test. Categorical variables were reported as frequency with percentage and analyzed using the chi-square test. The guidelines recommend the setting of an hsCRP level of 3 mg/L as a cutoff point for risk stratification of cardiovascular disease [17]. Therefore, we used an hsCRP cutoff of 3 mg/L to define elevated hsCRP levels. Increased PTFV1 was defined as > 5000 μV·ms according to a previous study [18]. The risk of outcomes was evaluated using Kaplan–Meier survival analysis with the log-rank test. Multivariable Cox proportional hazards models were used to assess the relationship between PTFV1 and outcomes. Models were adjusted for age, sex, NIHSS score, hypertension, diabetes mellitus, dyslipidemia, status as a current tobacco smoker, heart failure, coronary artery disease, stroke, TIA, medication (antiplatelets, anticoagulants, antihypertensive agents, hypoglycemic agents, and lipid-lowering agents), and baseline leukocyte count. A two-sided *p* < 0.05 was considered indicative of statistical significance. All data were analyzed using SAS statistical software, version 9.4 (SAS Institute, Inc., Cary, NC, USA).

## 3. Results

### 3.1. Patient Characteristics

After excluding patients with AF, a total of 8271 patients with both hsCRP and PTFV1 measurements were included in this analysis (Figure S1). Baseline characteristics were presented according to the PTFV1 threshold (Table 1). The median age of the participants was 62 years, and 68.6% of patients were men. The median hsCRP level was 1.6 mg/L (interquartile range, 0.8–4.2 mg/L). Elevated PTFV1 was associated with older age and higher hsCRP levels, NIHSS scores, and BMI. Additionally, there was a higher prevalence of males; histories of stroke, diabetes, hypertension, coronary heart disease, and heart failure; the usage of anticoagulants and antihypertensive agents; and the nonuse of antiplatelet agents among those with PTFV1 > 5000 μV·ms. The baseline characteristics and clinical features of the included and excluded participants are presented in Table S1. The excluded patients tended to be older and had higher prevalence values in terms of histories of coronary heart disease and heart failure and the usage of anticoagulants, while they had lower prevalence of smoking, histories of diabetes and dyslipidemia, and the usage of antiplatelet agents, hypoglycemic agents, and lipid-lowering agents. We adjusted these factors in multivariable cox proportional hazards models.

### 3.2. Associations of PTFV1 with Mortality According to hsCRP Levels

**Figure 1:**
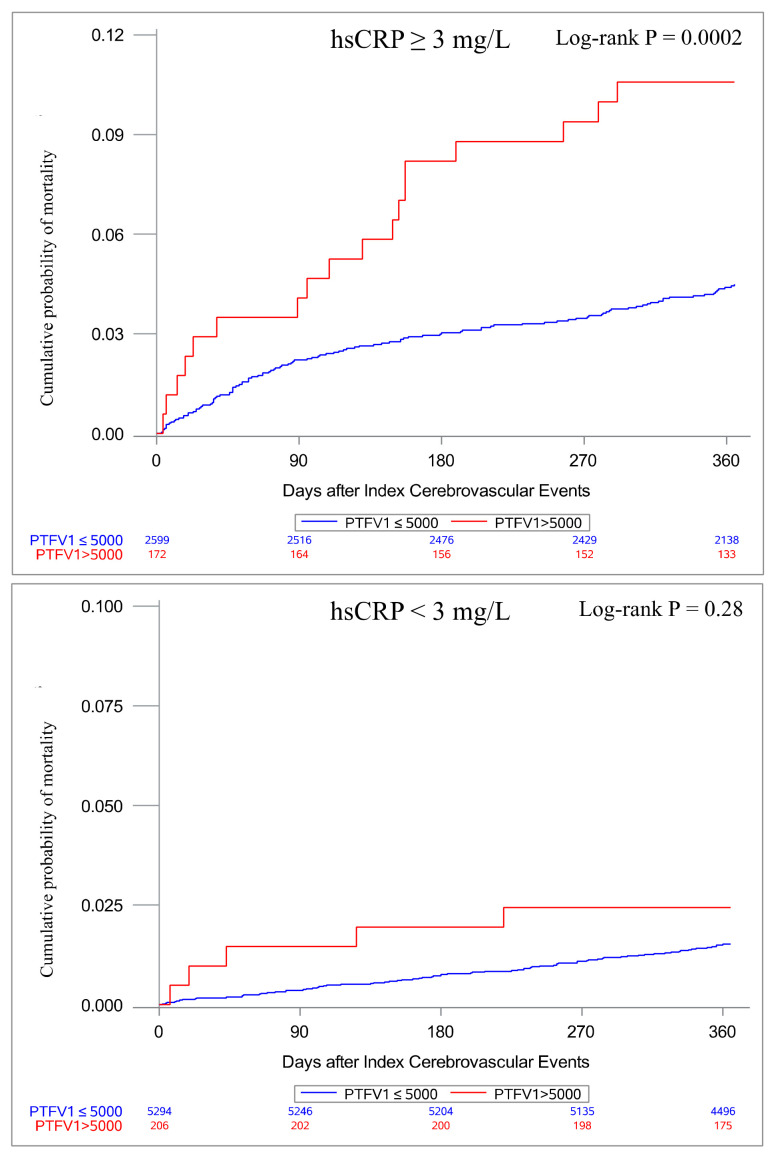
Kaplan–Meier curves for probability of mortality classified by hsCRP level. hsCRP, high-sensitivity C-reactive protein; PTFV1, P-wave terminal force in lead V1.

In total, 216 (2.6%) participants died within 1 year. PTFV1 > 5000 μV·ms was significantly associated with mortality (adjusted HR, 1.58; 95% CI, 1.01–2.47; *p* = 0.04) after adjustment. When classifying the patients according to their hsCRP levels, among patients with hsCRP levels ≥ 3 mg/L, elevated PTFV1 was significantly associated with mortality (adjusted HR, 1.75; 95% CI, 1.05–2.92; *p* = 0.03); among those with hsCRP levels < 3 mg/L, such an association did not exist (adjusted HR, 1.00; 95% CI, 0.39–2.56; *p* = 0.99) (Table 2). Figure 1 displays the cumulative incidence of mortality according to the hsCRP threshold. In those with hsCRP levels < 3 mg/L, PTFV1 > 5000 μV·ms was not associated with a higher cumulative incidence of mortality (*p* = 0.28). However, in those with hsCRP levels ≥ 3 mg/L, the cumulative incidence of mortality was significantly higher in the PTFV1 > 5000 μV·ms group (*p* = 0.0002).

### 3.3. Associations of PTFV1 with Ischemic Stroke Recurrence According to hsCRP Levels

**Figure 2:**
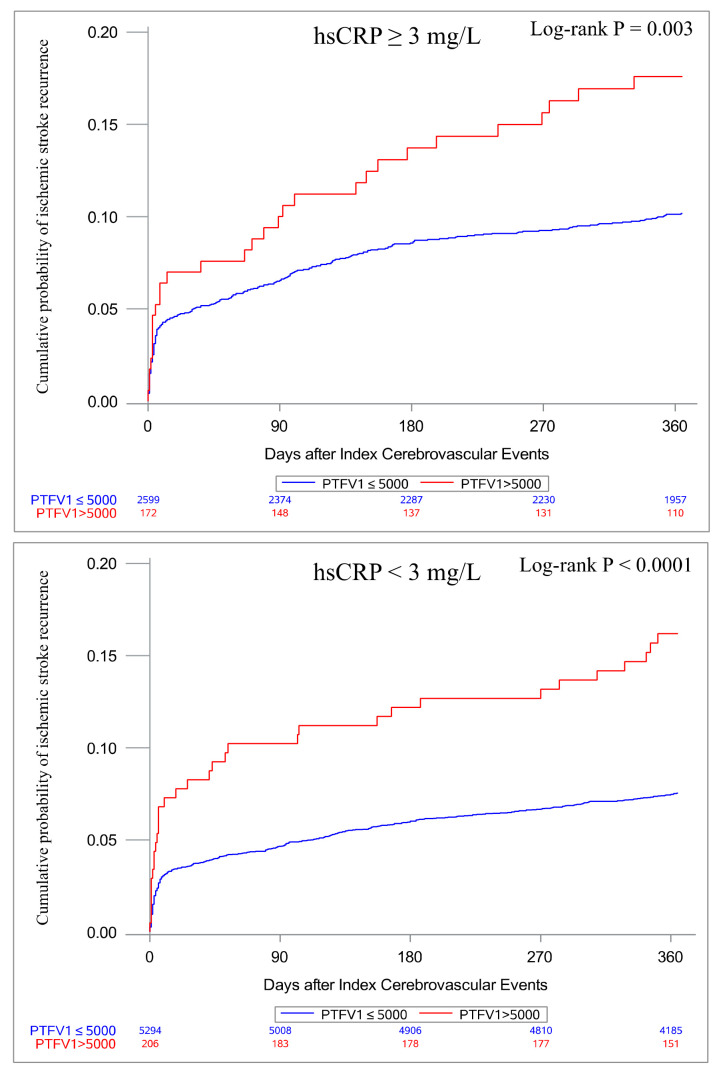
Kaplan–Meier curves for probability of ischemic stroke recurrence classified by hsCRP. hsCRP, high-sensitivity C-reactive protein; PTFV1, P-wave terminal force in lead V1.

In total, 715 (8.6%) participants experienced ischemic stroke recurrence within 1 year. Patients with PTFV1 > 5000 μV·ms had a significantly higher risk of ischemic stroke recurrence (adjusted HR, 1.86; 95% CI, 1.42–2.44; *p* < 0.0001) after adjustment. In patients with hsCRP levels < 3 mg/L (adjusted HR, 1.95; 95% CI, 1.35–2.82; *p* = 0.0004) and those with hsCRP levels ≥ 3 mg/L (adjusted HR, 1.74; 95% CI, 1.18–2.56; *p* = 0.005), PTFV1 > 5000 μV·ms remained significantly associated with ischemic stroke recurrence (Table 3). Figure 2 displays the cumulative incidence of ischemic stroke recurrence according to the hsCRP threshold. In both patients with hsCRP levels < 3 mg/L (*p* < 0.0001) and those with hsCRP levels ≥ 3 mg/L (*p* = 0.003), PTFV1 > 5000 μV·ms was associated with a higher cumulative incidence of ischemic stroke recurrence.

### 3.4. Associations of hsCRP with Prognosis According to PTFV1 Threshold

Patients with hsCRP levels ≥ 3 mg/L had a significantly higher risk of death (adjusted HR, 2.09; 95% CI, 1.56–2.80; *p* < 0.0001) and ischemic stroke recurrence (adjusted HR, 1.24; 95% CI, 1.06–1.45; *p* = 0.006) after adjustment (Tables S2 and S3). In patients with PTFV1 > 5000 μV·ms, an increased hsCRP level still showed a trend of a higher risk of death (adjusted HR, 2.75; 95% CI, 0.91–8.29; *p* = 0.07) (Table S2), while no significant association was observed between increased hsCRP levels and ischemic stroke recurrence (adjusted HR, 0.92; 95% CI, 0.53–1.62; *p* = 0.78) (Table S3).

## 4. Discussion

In this study, we found that elevated PTFV1 was associated with a higher risk of death and ischemic stroke recurrence among patients who had suffered an acute ischemic stroke or TIA and in the absence of AF. Furthermore, a PTFV1-associated mortality risk was only observed among patients with high hsCRP levels. The association between PTFV1 and ischemic stroke recurrence was not influenced by hsCRP levels.

AF is the primary cause of cardioembolism, accounting for 15% to 24% of ischemic strokes. A stroke resulting from AF is typically more severe, more likely to recur, and nearly twice as likely to be fatal as non-AF strokes [19]. Thrombus formation in patients with AF is caused by decreased LA appendage flow velocity, the activation of the coagulation cascade, and left atrial enlargement and fibrosis. Even short subclinical episodes of AF are linked to stroke, but the causal connection between AF and ischemic stroke remains indirect. Prior research has demonstrated that in older patients with vascular risk factors, a single brief episode of subclinical AF is linked to a higher risk of stroke [20]. Conversely, males with a low risk for stroke and clinically apparent AF do not face a significantly increased stroke risk [21]. These conflicting findings are insufficient with respect to establishing a definitive biological gradient between the burden of AF and the risk of stroke. It was reported that AF events were detected in only 8% of stroke patients within 30 days prior to the onset of a stroke, while 16% of stroke patients experienced their first AF event after the occurrence of stroke [22], suggesting that AF itself may not be the direct cause of stroke. Instead, it may be a risk indicator for embolic stroke due to underlying atrial dysfunction.

Recent advancements in pathophysiology suggest that left atrial degeneration, including chamber dilation, remodeling, fibrosis, and damage to endothelial cells and cardiomyocytes, can cause thrombus generation and embolism, even in patients without AF. This condition is referred to as atrial cardiopathy, which describes the structural and functional disorders of the atrium that can precede AF [1]. The abnormal atrial tissue substrate results from aging and systemic vascular risk factors, which increase the risk of AF and thromboembolism. Once AF develops, it causes contractile dysfunction and stasis, leading to a greater risk of thromboembolism. Moreover, the arrhythmia causes the structural remodeling of the atrium, further worsening atrial cardiopathy and increasing the risk of thromboembolism. Autonomic changes and inflammation post-stroke may transiently increase AF risk after stroke [23]. Rather than considering AF as the sole cause of thromboembolic risk in patients with AF, it is more helpful to perceive both AF and thromboembolism as common manifestations of an underlying atrial cardiopathy. The driving force of thromboembolism is not merely the arrhythmia but also a host of underlying pathological tissue changes. Nonetheless, AF remains a crucial factor with respect to thromboembolic risk because the arrhythmia worsens both the tissue changes and left atrial contractile function. While no established criteria exist for diagnosing atrial cardiopathy, researchers have tried to identify biomarkers associated with stroke to detect the condition early. One crucial factor that connects atrial cardiopathy and AF with stroke is inflammation [12]. Patients with AF have increased levels of inflammatory markers such as CRP, tumor necrosis factor-α, and interleukin-2, -6, and -8 [14]. The Women’s Health Study demonstrated that in women without a history of cardiovascular disease, inflammatory biomarkers, including CRP, soluble intercellular adhesion molecule-1, and fibrinogen, were independently associated with an increased incidence of AF, even after controlling for traditional risk factors [24]. Among AF patients, CRP was positively correlated with stroke risk and related to stroke risk factors and prognosis (mortality and vascular events) [25].

Previous studies have suggested that there is a positive association between PTFV1 and sudden cardiac death, cardiovascular death [26], and all-cause death [4], independent of clinical cardiovascular risk factors. Our results proved the independent predictive role of PTFV1 with respect to mortality after stroke and further added evidence regarding the effect of hsCRP on it. We found that PTFV1 was associated with mortality only in those with elevated hsCRP levels. Many factors may account for the association between PTFV1 and mortality. First, PTFV1 reflects atrial changes, such as left atrial hypertrophy and interatrial conduction defects [27], and its increase has been associated with impaired left atrial function and possibly eventual mortality [28,29]. Second, a recent study confirmed an association between abnormal PTFV1 and left ventricular fibrosis determined via cardiac MRI [28]. In addition, PTFV1 has been suggested to be an early manifestation of left ventricular diastolic dysfunction [30], which is an independent predictor of mortality [31]. Third, PTFV1 was significantly associated with brain vascular injury and infarction [32], which might lead to an increased risk of mortality. On the other hand, inflammation has also been indicated to be involved in the dysfunction of the left atrium and left ventricle [12,33] and brain infarction [34,35]. Therefore, patients with elevated PTFV1 and hsCRP levels are likely to be exposed to greater abnormalities correlated with mortality. Furthermore, in view of hsCRP itself being a direct independent factor of mortality after stroke, through various mechanisms other than those aforementioned [36], hsCRP may play a more important role in predicting mortality than PTFV1. This might be one possible explanation for the absence of an association of PTFV1 with mortality in patients with elevated PTFV1 but low hsCRP levels found in our study.

Furthermore, our findings expand the previous data on the relationship between PTFV1 and stroke in the absence of AF. Atrial thromboembolism caused by PTFV1 has been suggested to contribute to the incidence of stroke [5]. Although inflammation plays a role in not only thrombosis but also atherosclerotic plaque development and rupture [37], we found that PTFV1-associated ischemic stroke recurrence was not affected by hsCRP levels, suggesting a dominant role of PTFV1 in recurrent stroke. The results indicate that PTFV1 has a stronger association with recurrent stroke than with mortality, which could be partly explained by hsCRP being an overall indicator of poor prognosis, while PTFV1 was more specifically associated with the pathophysiology of ischemic stroke. Taken together, our findings, to some extent, indicated different mechanisms that lead to PTFV1’s ability to cause recurrent stroke and mortality and helped stratify the risk of recurrent stroke and mortality by incorporating hsCRP.

The most clinically evident biomarker of atrial cardiopathy is AF. However, it seemed likely that atrial cardiopathy evolved long before AF became clinically evident. PTFV1 was significantly associated with the risk of AF [38,39]. Inflammation was shown to be involved in the inception, recurrence, and perpetuation of AF [40]. In addition, it contributed to promoting a prothrombotic AF state via endothelial dysfunction, platelet activation, and the increased expression of fibrinogen [14]. Inflammatory cells, such as lymphocytes, monocytes, and macrophages, are responsible for producing cytokines and chemokines, which can trigger thrombosis in AF. IL-6 has been found to stimulate the expression of tissue factor, fibrinogen, factor VIII, and von Willebrand factor, thereby promoting a pro-thrombotic state. IL-6 may also cause endothelial activation and endothelial cell damage, leading to platelet aggregation and increased sensitivity to thrombin [14]. Activated platelets can further promote and maintain the pro-thrombotic state while also increasing the levels of inflammatory biomarkers in patients with AF. In addition to these effects, altered endothelial function can also contribute to inflammation and thrombosis in AF. Endothelial activation leads to the rapid release of substances, such as von Willebrand factor and soluble P-selectin, onto the endothelial surface, which promote the attachment of rolling white blood cells to the endothelium and contribute to the development of a pro-inflammatory and pro-thrombotic environment [12]. It is possible that this inflammatory state contributes to the development of atrial cardiopathy, which leads to endothelial dysfunction [13] and atrial structural changes [15] and results in a greater state of inflammation. The underlying pathophysiology represented by these biomarkers may provide insight into atrial cardiopathy. We excluded patients with AF since the association between AF and stroke prognosis was clear. The current study focused on patients with manifestations of atrial cardiopathy before the occurrence of AF.

This study has some limitations that should be considered when interpreting the results. First, the exclusion of patients with incomplete markers or AF might have introduced potential selection bias. Second, the manual measurement of PTFV1 rather than automated measurements might have limited the accuracy of the results. Nevertheless, the use of independent investigators for the blinded measurements enhanced the objectivity and reproducibility of the data. Third, this study was conducted in a Chinese population and may not be directly generalizable to other populations with different characteristics. Finally, although our findings suggest an association between PTFV1, hsCRP, and the prognosis of patients who have suffered an ischemic stroke or TIA, the observational nature of the study precludes our ability to infer a causal relationship. Further experimental and randomized studies are necessary to confirm whether PTFV1–hsCRP interaction has a causal effect on the prognosis of patients who have suffered an ischemic stroke or TIA.

## 5. Conclusions

In patients who have suffered an acute ischemic stroke or TIA but without AF, PTFV1-associated mortality risk was more apparent in patients with high hsCRP levels, while the association between PTFV1 and ischemic stroke recurrence was not influenced by hsCRP levels.

## Figures and Tables

**Table 1:** Baseline characteristics of the study population classified by PTFV1.

Characteristics	Total (N = 8271)	PTFV1 > 5000 μV·ms (n = 378)	PTFV1 ≤ 5000 μV·ms (n = 7893)	*p* Value
Age, y, median (IQR)	62 (54–69)	64 (56–72)	62 (53–69)	<0.0001
Male, n (%)	5678 (68.6)	294 (77.8)	5384 (68.2)	<0.0001
Body mass index, kg/m^2^, median (IQR)	24.5 (22.6–26.6)	25.1 (23.4–27.4)	24.5 (22.6–26.6)	<0.0001
Smoking, n (%)	2687 (32.5)	110 (29.1)	2577 (32.6)	0.15
Medical history, n (%)				
Stroke	1872 (22.6)	110 (29.1)	1762 (22.3)	0.002
TIA	244 (2.9)	7 (1.8)	237 (3.0)	0.20
Diabetes	2000 (24.2)	117 (30.9)	1883 (23.9)	0.002
Hypertension	5197 (62.8)	294 (77.8)	4903 (62.1)	<0.0001
Dyslipidemia	695 (8.4)	42 (11.1)	653 (8.3)	0.052
Coronary heart disease	803 (9.7)	71 (18.8)	732 (9.3)	<0.0001
Heart failure	30 (0.4)	10 (2.6)	20 (0.2)	<0.0001
Baseline NIHSS score, median (IQR)	3 (1–6)	4 (2–7)	3 (1–6)	<0.0001
Leukocyte count, *109/L, median (IQR)	6.9 (5.7–8.3)	7.0 (5.7–8.5)	6.8 (5.7–8.3)	0.47
hsCRP, mg/L, median (IQR)	1.6 (0.8–4.2)	2.6 (1.2–6.5)	1.6 (0.8–4.1)	<0.0001
Medication, n (%)				
Antiplatelet agent	7832 (94.7)	341 (90.2)	7491 (94.9)	<0.0001
Anticoagulants	51 (0.6)	8 (2.1)	43 (0.5)	0.0001
Antihypertensive agent	4069 (49.2)	234 (61.9)	3835 (48.6)	<0.0001
Hypoglycemic agent	2051 (24.8)	109 (28.8)	1942 (24.6)	0.06
Lipid-lowering agent	7703 (93.1)	345 (91.3)	7358 (93.2)	0.14

hsCRP, high-sensitivity C-reactive protein; IQR, interquartile range; NIHSS, National Institutes of Health Stroke Scale; PTFV1, P-wave terminal force in lead V1.

**Table 2:** Associations between PTFV1 and mortality within 1 year according to hsCRP threshold.

Groups	Events/Total (%)		HR (95% CI)	*p* Value
Total				
PTFV1 ≤ 5000 μV·ms	193/7893 (2.4)		reference	NA
PTFV1 > 5000 μV·ms	23/378 (6.1)	Model 1	1.72 (1.10–2.67)	0.02
		Model 2	1.58 (1.01–2.47)	0.04
hsCRP < 3 mg/L				
PTFV1 ≤ 5000 μV·ms	79/5294 (1.5)		reference	NA
PTFV1 > 5000 μV·ms	5/206 (2.4)	Model 1	1.23 (0.49–3.08)	0.66
		Model 2	1.00 (0.39–2.56)	0.99
hsCRP ≥ 3 mg/L				
PTFV1 ≤ 5000 μV·ms	114/2599 (4.4)		reference	NA
PTFV1 > 5000 μV·ms	18/172 (10.5)	Model 1	1.82 (1.09–3.04)	0.02
		Model 2	1.75 (1.05–2.92)	0.03

HR, hazard ratio; hsCRP, high-sensitivity C-reactive protein; PTFV1, P-wave terminal force in lead V1; NA, not available. Model 1: age, sex, NIHSS score, hypertension, diabetes mellitus, dyslipidemia, current tobacco smoker status, heart failure, coronary artery disease, stroke, and TIA. Model 2: Model 1 + medication (antiplatelets, anticoagulants, antihypertensive agents, hypoglycemic agents, and lipid-lowering agents) + baseline leukocyte count.

**Table 3:** Associations of PTFV1 with ischemic stroke recurrence within 1 year according to hsCRP threshold.

Groups	Events/Total (%)		HR (95% CI)	*p* Value
Total				
PTFV1 ≤ 5000 μV·ms	653/7893 (8.3)		reference	NA
PTFV1 > 5000 μV·ms	62/378 (16.4)	Model 1	1.93 (1.48–2.52)	<0.0001
		Model 2	1.86 (1.42–2.44)	<0.0001
hsCRP < 3 mg/L				
PTFV1 ≤ 5000 μV·ms	394/5294 (7.4)		reference	NA
PTFV1> 5000 μV·ms	33/206 (16.0)	Model 1	2.10 (1.46–3.01)	<0.0001
		Model 2	1.95 (1.35–2.82)	0.0004
hsCRP ≥ 3 mg/L				
PTFV1 ≤ 5000 μV·ms	259/2599 (10.0)		reference	NA
PTFV1 > 5000 μV·ms	29/172 (16.9)	Model 1	1.76 (1.19–2.60)	0.004
		Model 2	1.74 (1.18–2.56)	0.005

HR, hazard ratio; hsCRP, high-sensitivity C-reactive protein; PTFV1, P-wave terminal force in lead V1; NA, not available. Model 1: age, sex, NIHSS score, hypertension, diabetes mellitus, dyslipidemia, current tobacco smoker status, heart failure, coronary artery disease, stroke, and TIA. Model 2: Model 1 + medication (antiplatelets, anticoagulants, antihypertensive agents, hypoglycemic agents, and lipid-lowering agents) + baseline leukocyte count.

## Data Availability

All data are available to researchers by contacting the corresponding author.

## Supplementary Materials

The following supporting information can be downloaded at: https://www.mdpi.com/article/10.3390/jcm12052031/s1, Figure S1: Flow chart showing the participants’ selection; Table S1: Baseline characteristics of patients included versus not included; Table S2. Associations of hsCRP with mortality within 1 year according to PTFV1 threshold; Table S3. Associations of hsCRP with ischemic stroke recurrence within 1 year according to PTFV1 threshold.
