# Supplementary material for: High-Sensitivity C-Reactive Protein Modifies P-Wave Terminal Force in Lead V1-Associated Prognosis in Acute Ischemic Stroke or TIA Patients
Source: J Clin Med. 2023 Mar 3;12(5):2031. doi: 10.3390/jcm12052031 (PMC10003915; doi:10.3390/jcm12052031)
Supplement: Supplementary file 1 [file jcm-12-02031-s001.zip › jcm-2236154-supplementary.pdf]

Table S1. Baseline characteristics of patients included versus not included.

Characteristics	Patients included (n = 8271)	Not included (n = 6895)	P value
Age, y, median (IQR)	62 (54-69)	63 (55-71)	< 0.0001
Male, n (%)	5678 (68.6)	4686 (68.0)	0.36
Body mass index, kg/m2, median (IQR)	24.5 (22.6-26.6)	24.5 (22.6-26.4)	0.03
Smoking, n (%)	2687 (32.5)	2065 (29.9)	0.0008
Medical history, n (%)			
Stroke	1872 (22.6)	1483 (21.5)	0.10
TIA	244 (2.9)	172 (2.5)	0.09
Diabetes	2000 (24.2)	1510 (21.9)	0.0009
Hypertension	5197 (62.8)	4297 (62.3)	0.51
Dyslipidemia	695 (8.4)	496 (7.2)	0.006
Coronary heart disease	803 (9.7)	805 (11.7)	< 0.0001
Heart failure	30 (0.4)	64 (0.9)	< 0.0001
Baseline NIHSS score, median (IQR)	3 (1-6)	3 (1-6)	0.003
Leukocyte count, *109/L, median (IQR)	6.9 (5.7-8.3)	7.0 (5.7-8.5)	0.003
Medication, n (%)			
Antiplatelet agent	7832 (94.7)	5879 (85.3)	< 0.0001
Anticoagulants	51 (0.6)	398 (5.8)	< 0.0001
Antihypertensive agent	4069 (49.2)	3349 (48.6)	0.44
Hypoglycemic agent	2051 (24.8)	1495 (21.7)	< 0.0001
Lipid-lowering agent	7703 (93.1)	6128 (88.9)	< 0.0001

hsCRP, high-sensitivity C-reactive protein; IQR, interquartile range; NIHSS, National Institutes of Health Stroke Scale.

Table S2. Associations of hsCRP with mortality within 1 year according to PTFV1 threshold.

Groups	Events/Total (%)		HR (95% CI)	*p* value
Total				
hsCRP < 3 mg/L	84/5500 (1.5)		reference	NA
hsCRP ≥ 3 mg/L	132/2771 (4.8)	Model 1	2.07 (1.55-2.77)	< 0.0001
		Model 2	2.09 (1.56-2.80)	< 0.0001
PTFV1 ≤ 5000μV·ms				
hsCRP < 3 mg/L	79/5294 (1.5)		reference	NA
hsCRP ≥ 3 mg/L	114/2599 (4.4)	Model 1	1.96 (1.45-2.66)	< 0.0001
		Model 2	1.96 (1.44-2.67)	< 0.0001
PTFV1 > 5000μV·ms				
hsCRP < 3 mg/L	5/206 (2.4)		reference	NA
hsCRP ≥ 3 mg/L	18/172 (10.5)	Model 1	2.81 (0.97-8.18)	0.06
		Model 2	2.75 (0.91-8.29)	0.07

HR, hazard ratio; hsCRP, high-sensitivity C-reactive protein; PTFV1, P-wave terminal force in lead V1; NA, not available. Model 1: age, sex, NIHSS score, hypertension, diabetes mellitus, dyslipidemia, current tobacco smoker status, heart failure, coronary artery disease, stroke, and TIA. Model 2: Model 1 + medication (antiplatelets,

anticoagulants, antihypertensive agents, hypoglycemic agents, and lipid-lowering agents) + baseline leukocyte count.

Table S3. Associations of hsCRP with ischemic stroke recurrence within 1 year according to PTFV1 threshold.

Groups	Events/Total (%)		HR (95% CI)	*p* value
Total				
hsCRP < 3 mg/L	427/5500 (7.8)		reference	NA
hsCRP ≥ 3 mg/L	288/2771 (10.4)	Model 1	1.25 (1.07-1.46)	0.005
		Model 2	1.24 (1.06-1.45)	0.006
PTFV1 ≤ 5000μV·ms				
hsCRP < 3 mg/L	394/5294 (7.4)		reference	NA
hsCRP ≥ 3 mg/L	259/2599 (10.0)	Model 1	1.26 (1.07-1.48)	0.006
		Model 2	1.25 (1.06-1.47)	0.007
PTFV1 > 5000μV·ms				
hsCRP < 3 mg/L	33/206 (16.0)		reference	NA
hsCRP ≥ 3 mg/L	29/172 (16.9)	Model 1	1.00 (0.58-1.70)	0.99
		Model 2	0.92 (0.53-1.62)	0.78

HR, hazard ratio; hsCRP, high-sensitivity C-reactive protein; PTFV1, P-wave terminal force in lead V1; NA, not available. Model 1: age, sex, NIHSS score, hypertension, diabetes mellitus, dyslipidemia, current tobacco smoker status, heart failure, coronary artery disease, stroke, and TIA. Model 2: Model 1 + medication (antiplatelets, anticoagulants, antihypertensive agents, hypoglycemic agents, and lipid-lowering agents) + baseline leukocyte count.

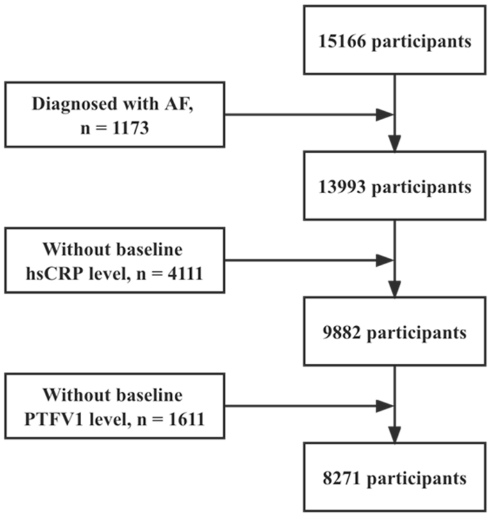

Figure S1. Flow chart showing the participants' selection. AF, atrial fibrillation; hsCRP, high-sensitivity C-reactive protein; PTFV1, P-wave terminal force in lead V1.
